# Assessing the Effect of Rural Placements Through a Pre‐Post Approach With Different Temporal Targets: Are We Converting or Sustaining?

**DOI:** 10.1111/ajr.70176

**Published:** 2026-04-10

**Authors:** Claire Ellen Seaman, Elyce Green

**Affiliations:** ^1^ Three Rivers Department of Rural Health Charles Sturt University Wagga Wagga New South Wales Australia

## Abstract

**Background:**

Rural placements are a key strategy to attract health students to rural practice. It is important that robust research methods are used to assess the effect of placements on rural career intentions to inform resource allocation and student supports.

**Objectives:**

This study explored the utility of a novel rural attitude measurement approach to evaluate the direct impact of rural placements on students' perceptions and intentions. It also examined whether anticipated differences between students from city and rural backgrounds emerged across various future temporal reference points. Finally, the research investigated the characteristics of students who demonstrated desirable rural placement outcomes, specifically those who either ‘converted’ to or ‘sustained’ an early career attraction to rural practice.

**Method:**

This research employed a pre‐post cohort design, surveying health students who were commencing an Australian rural placement. Participants included those from Charles Sturt University as well as students from other universities receiving support from Three Rivers Department of Rural Health. A total of 163 matched pre‐ and post‐placement surveys were collected. The survey measured ratings of the attractiveness of living and working outside of a Major City area across two future timepoints: (1) during the early years of practice (1–3 years after graduation) and (2) after establishing a career (10–15 years after graduation), using an 11‐point scale.

**Results:**

Most respondents were from a rural background (78%), 29% had care responsibilities, and 36% were aged 30 years and older. Overall, only mid‐career attraction significantly increased from pre‐placement levels; however, important differences were identified among subgroups.

**Conclusions:**

Rural placement experiences serve a ‘sustaining’ function for those already committed to, and likely located in, rural areas. The desired ‘converting’ effect of rural placements may be constrained under current RHMT Program scope for health students, especially those from city areas.

## What Is Already Known on the Subject

– Rural placements are a major rural workforce attraction activity with the expectation they will lead to increased interest in rural practice among students and actualised rural practice when they graduate.
– Retrospective cross‐sectional measures suggest rural placements are most effective for converting students with limited rural experience (i.e., city‐background).
– Rural background is the predominant factor in future rural practice outcomes.

**What this paper adds**:

– A pre‐post longitudinal assessment of the effect of rural placements on attraction to living and working rurally at two future career timepoints.
– Taking a novel approach to examine what ‘sustains’ and ‘converts’ students' attraction to early career rural practice, the research suggests positive rural exposure through placements must be considered concomitant with regional mobility factors and associated drivers, especially for realising conversions.
– The persistent ceiling effects found in measures of rural work attitudes among a predominately regional university sample highlight the importance of studying rurally. There is a need to focus research and policy on the mobility effects of studying in regional areas beyond placement experiences.

## 1 Introduction

Rural placements are a key component of efforts to strengthen recruitment of allied health, nursing, midwifery, dentistry, and oral health professionals into Australia's rural health workforce, alongside other complementary strategies [1, 2, 3]. Underscoring this approach is the expectation that rural placements enhance students' attitudes towards living and working rurally and thereby increase the likelihood they will choose to do so when they graduate. This expectation has led to extensive resource and financial investment, notably as part of the Rural Health Multidisciplinary Training (RHMT) Program [4, 5]. Due to the significance of this investment, there is a need to conduct methodologically rigorous research to determine whether rural placements influence rural practice attitudes and for what career time points this outcome is observed [6]. This study aimed to address this gap using a pre‐post‐placement survey of students' attraction to living and working rurally given two future temporal reference points: early career (1–3 years after graduating) and mid‐career (10–15 years of practice).

## 2 Background

Rural workforce maldistribution is a regional mobility issue and, reflecting this, conceptual work has been undertaken to apply mobility and place attachment approaches to rural health workforce studies [7, 8, 9, 10, 11, 12, 13]. There is a need for more direct engagement with a migration lens in health workforce studies beyond medicine [7]. Migration approaches view individual and household mobility or immobility decision‐making as being an active negotiation of perceived barriers, enablers, risks and benefits of living where and when, with decisions and place attachment commonly patterned by life course stages [14, 15]. For instance, in Australia, moving to engage in tertiary education is most common among those in early adulthood [9]. More broadly, singles and households without children are notable mobile groups, while dual‐earner dual‐degree qualified households are a key group unlikely to engage in long‐distance mobility in Australia [16]. This reflects recent findings from rural health workforce research showing partners' employment and proximity to family and friends as key factors in health graduates from two Major City‐area Australian universities choosing a city work location [17].

In Australia, a primary activity of University Departments of Rural Health (UDRHs) under the RHMT Program is the delivery of longer, full‐time rural health student placements to attract future health workers to rural areas [4]. The RHMT Program's support for these rural placements implicitly functions with the rationale of mobilising or converting students towards rural practice by providing immersive rural exposure (i.e., rural experiences, insights, and social ties) [18]. As such, there is limited direct examination of the expected effect of rural placements among students with already strong ties and intentions to work in rural communities.

Measures of the effect of rural placements on attitudes and intentions to future rural practice in Australin nursing and allied health contexts are characterised by ceiling effects [6]. Ceiling effects in this instance refers to substantial numbers of students rating rural attitudes and intentions at the highest possible category, which suggests these responses may be censored [19]. This is problematic because it may mask differences in improvements and can lead to inaccurate conclusions about the impact of placements. This may be a result of the limited measures used or due to a natural ceiling effect whereby students' attitudes cannot be further enhanced. There are key student groups among whom this is expected to occur. For example, the social and financial ties of rural mature‐aged students mean they are highly likely to practice in their existing communities [20, 21]. Glenister and colleagues [21] specifically examined differences among rural‐ and city‐background students, as well as by whether these groups were studying at universities located in or outside city areas. Their results demonstrated the converting effect of placements was limited for rural‐background students because they already had higher intentions to practice rurally, with even city‐background students studying outside of a city‐area having higher retrospectively assessed pre‐placement rural practice intentions than their city‐studying counterparts. For students studying regionally, repeated short‐term placements have also been shown to have a cumulative effect to enhance future rural practice intentions among city‐background students [22]. A positive effect of cumulative rural placements on future rural practice has been indicated by other Australian studies [23, 24].

Overall, this demonstrates the need for a greater understanding of both the expected converting and sustaining effect of placements, based on rurality of background as well as pre‐placement attitudes. Australian research examining the direct effect of placements on rural work attitudes and intentions has predominately relied on post‐placement cross‐sectional measures, and often with responses to a five‐point Likert scale statement where ceiling effects are substantial [6]. The statement, ‘This placement has encouraged me to consider living and working in a rural area when I graduate’, or similar is commonly used in UDRH studies [6, 21]. While this measure specifies the immediate post‐graduate time period, there is the potential that students' responses are also reflective of an unobserved change in practice attitudes at a later time period. This requires further examination as Playford and colleagues [25] found rural placements to be associated only with early‐career rural practice and not later‐career practice. Thus, in measuring the effect of placement, emphasis should be on early‐career, as in current measures, however, inclusion of a later‐career point of comparison may reduce measurement bias from a single measure of attitudes towards rural practice [26].

This study aimed to address these gaps using a longitudinal pre‐post design with an elongated scale of rural living and working attraction, assessed against two distinct temporal targets. In doing so, this study sought to make three important contributions to the literature. The first was by examining how rural placement experiences influence attraction to rural practice across early and mid‐career stages, offering a longitudinal perspective rarely explored in previous research. Secondly, this study adds nuance by comparing city‐ and rural‐background students, highlighting differential impacts of placement on career intentions. Finally, by identifying and characterising two key outcome groups–‘converters,’ who shift towards rural practice post‐placement, and ‘sustainers,’ who maintain strong rural attraction–the study provides actionable insights for targeted workforce strategies. This reflects a focus from migration literature that seeks to understand characteristics and motivations of ‘movers’ and ‘stayers’ [14, 27].

## 3 Research Questions

This study addressed three research questions:

1. What is the distribution and change in attractiveness to rural practice in early‐ and mid‐career, given a rural placement experience?
2. What are the differences for city‐ and rural‐background students?
3. What are the demographic and placement characteristics of the ideal outcome groups, (a) ‘converters’ to, and (b) ‘sustainers’ of, high early career rural practice attraction?

## 4 Methods

This study used a pre‐post survey design. Data was collected before and after a rural placement experience to examine changes in attitudes towards rural practice.

### 4.1 Recruitment

Students were identified using the university's placement management system and UDRH placement support registers. The researchers emailed students and invited them to complete an online survey prior to starting their placement. Between September 2018 and June 2020, students undertaking 958 placements were emailed to request their participation in the study. While the systematic recruitment messaging was sent directly to Charles Sturt University students, all students on rural placements were eligible to complete a survey (including students enrolled at other universities), and the survey was broadly publicised. Any student who completed the survey before their placement was emailed again after their placement to complete a follow‐up. In the pre‐placement survey, respondents were given the opportunity to request a follow‐up text message, which was sent in addition to the email.

### 4.2 Survey Instrument

An online survey was administered in two parts under the ‘MyPlacement’ project at Charles Sturt University, a regional university with six major campuses across regional New South Wales: (1) a pre‐placement survey to be completed prior to commencement of a rural placement; and (2) a post‐placement survey to be completed when the rural placement was finished. Students were allocated the appropriate survey and questions based on their response to screening questions that asked whether they had completed a ‘MyPlacement’ survey previously, and whether they were about to start or had finished their referent rural placement.

At the start of the survey, a broad rural definition was provided for students as, 'anywhere in Australia outside of Adelaide, Brisbane, Melbourne, Perth, Sydney, Wollongong, Newcastle, Geelong, the Sunshine Coast, and the Gold Coast per the ‘Inner Regional’ to ‘Very Remote’ area classification of the Australian Bureau of Statistics' Australian Statistical Geographic Standard Remoteness Areas (ASGS‐RA 2016). The pre‐post measure included in both surveys asked students to rate their attractiveness to living and working outside of a Major City area at different stages of their career: early career (1–3 years after graduation), and after establishing their career (10–15 years after graduation). A range of questions about study choice, demographics, and placement experiences were included, building off existing surveys developed and administered across the UDRH network [21]. Pre‐post surveys were linked using either student numbers (for Charles Sturt University students) or a primary university email (for student from any other university).

### 4.3 Data Analysis

Paired t–tests were used to assess individual change in rural attraction scores at post‐placement from pre‐placement. While the response distributions violated distribution assumptions of linear regression modelling for some models, comparison with Wilcoxon‐signed rank tests yielded consistent results. For easier interpretation of the results relative to the original scale, and to avoid changing between model specifications, only the parametric results are presented. The results showed the latent phenomena appeared to be characterised by natural ceiling effects. To deal with this, overall pre‐post change scores are first presented, followed by change scores limited to those who scored below the median in the pre‐placement measures. Additionally, proportions of students who scored lower, higher or no change were calculated and presented, using a cut‐point of 2 scale points or greater to indicate change. To assess the differentiating characteristics of ‘converter’ and ‘sustainer’ students relative to the respective lower‐ and higher‐ pre‐placement early career attraction scores, chi‐square proportion tests were conducted for the variables resented in Table 1. Analyses where there were missing data followed listwise deletion of cases. Data cleaning and analysis was completed using Stata/SE 15.1.

**TABLE 1:** Student demographic and placement characteristics.

Demographic characteristics		
	*N*	%
Ever lived rurally 5+ years		
Yes	127	77.91
No	36	22.09
Gender		
Male	21	12.88
Female	142	87.12
Married or de facto		
No	109	66.87
Yes	54	33.13
Age (missing = 1)		
18–21	49	30.25
22–29	54	33.33
30+	59	36.42
Employment status		
Not in paid work	40	24.54
Working < 20 h/week	57	34.97
Working 20+ h/week	66	40.49
Care responsibilities		
No	115	70.55
Yes	48	29.45
Place of birth		
Other country	15	9.20
Australia	148	90.80
Study year level		
1	21	12.88
2	58	35.58
3	50	30.67
4	34	20.86
Study discipline		
Allied health	57	34.97
Nursing	106	65.03
Prior to this placement, I was considering living and working in a regional, rural or remote location following graduation (missing = 5)		
Strongly disagree	10	6.33
Somewhat disagree	13	8.23
Neither	26	16.46
Somewhat agree	54	34.18
Strongly agree	55	34.81

Placement characteristics		
	*N*	%
Placement length		
1 week or less	3	1.84
2 weeks	66	40.49
3–4 weeks	30	18.40
5–6 weeks	50	30.67
More than 6 weeks	14	8.59
Primary placement financial supports (multi select)		
Continued to work	25	15.34
Government support payments	50	30.67
Own savings/family support	129	79.14
Mortgage or credit card debt	8	4.91
Scholarship/TRDRH subsidised accommodation	20	12.27
Preference for allocated placement (missing = 3)		
First preference	33	20.63
Not first preference	38	23.75
No say/not any preference	89	55.63
Satisfied with placement workplace supervision (missing = 3)		
Strongly disagree	9	5.63
Disagree	17	10.63
Neither	16	10.00
Agree	46	28.75
Strongly agree	72	45.00
Overall, I was satisfied with my placement (missing = 5)		
Strongly disagree	4	2.53
Somewhat disagree	10	6.33
Neither	13	8.23
Somewhat agree	55	34.81
Strongly agree	76	48.10
Stayed in own home for placement (missing = 3)		
No	131	81.88
Yes	29	18.33
This placement has encouraged me to consider living and working in a rural or remote location after I graduate (missing = 8)		
Strongly disagree	12	7.69
Somewhat disagree	10	6.41
Neither	32	20.51
Somewhat agree	50	32.05
Strongly agree	52	33.33

### 4.4 Ethical Considerations

This project received Charles Sturt University Human Research Ethics Committee approval, project number H18189. Consent to participate in the surveys was embedded in the first page of the survey platform so respondents could read the participant information and consent details before commencing the survey.

## 5 Results

There were 438 respondents who completed at least one survey before or after their placement. Of these, 163 completed both a pre‐ and post‐placement survey and were included in the present analysis. The demographic and placement characteristics of these students are described in Table 1. More than three‐quarters (78%) of respondents identified as being from a rural background, 29% had dependent children or other care responsibilities, and 36% were aged 30 years and older. Students were most commonly on placement for five to six weeks (50%) or two weeks (40%), and most were placed without having a say or in their preferred locations (56%). Satisfaction with workplace supervision and the placement overall was high (74% and 83% agreement respectively).

### 5.1 What Is the Distribution and Change in Attractiveness to Rural Practice in Early‐ and Mid‐Career, Given a Rural Placement Experience?

The distribution of scores on the rural attractiveness ratings was more even than the five‐point scale but still with pronounced skew and ceiling effects. One‐quarter indicated the highest possible value of 10 when considering rural living and working in their early career at both pre– (24.22%) and post– (25.15%) placement surveys. Mid‐career rural attraction top‐scores were selected by 27.61% pre– and 28.22% post‐placement. The commonly used post‐placement assessment on a five‐point Likert scale for the statement, 'This placement encouraged me to consider living and working rurally after graduation', had a greater concentration of scores in the highest category, ‘Strongly agree’ (33.33%) and second‐highest category ‘Agree’ (32.05%). Only 7.69% strongly disagreed and 6.41% disagreed with this statement, with 20.51% selecting neither. The concomittant retrospective prior placement rural practice intention ratings were similarly distributed with 34.18% agreeing and 34.81% strongly agreeing.

**FIGURE 1:**
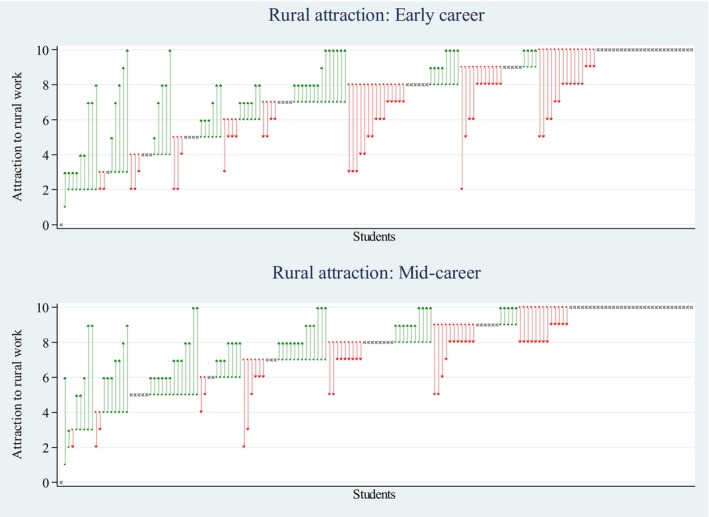
Distribution of pre‐ and post‐placement scores per student respondents. Red arrows show a negative change, green a positive change, and grey marks no change in scores.

The distribution of rural atractiveness responses pre‐placement and how each individuals' scores changed post‐placement are graphed in Figure 1, with red denoting a negative change, green a positive change and grey the same score. Overall, mean early‐career scores did not change from pre– (*M* = 7.23, SD = 2.53) to post‐placement (*M* = 7.23, SD = 2.46). Mean mid‐career scores significantly increased pre– (*M* = 7.55, SD = 2.28) to post (*M* = 7.87, SD = 2.04) by one‐third of a scale point. The median of both scales at pre‐ and post‐placement was 8. Examination of scores in early versus mid‐career based on a change of greater or less than 2 scale points indicated that the lack of change in early‐career attraction may be attributed to similar proportions of worse (20%) and improved (19%) scores. Conversely, just over one‐in‐ten (12%) had worse mid‐career rural attraction scores, while one‐in‐five (21%) improved. Most of the overall sample had no substantial change (< |1| scale points) in early– (61%) and mid‐career (68%) scores. Respondents who had a pre‐placement rural attraction score less than the median (8) had significantly improved scores of more than one scale point for early– (pre– *M* = 4.92, SD = 1.86, post– *M* = 6.09, SD = 2.48) and mid‐career scales (pre– *M* = 5.32, SD = 1.60, post– *M* = 6.57, SD = 2.11); however, the post‐placement mean and proportion improved were again descriptively higher for the mid‐career scale, this is also shown in Table 2.

**TABLE 2:** Changes in rural attraction scores post placement.

	Rural attraction scale	*N*	Pre mean.	Post mean.	Mean diff. (post‐pre).	Mean diff 95% CI	*T*	*p*	Worse (≤ 2), %.	No change (< |1|), %.	Improved (≥ 2), %.
*Overall*											
	Early career	163	7.23	7.23	0.00	[−0.35, 0.35]	0.00	1.00	20.25	60.74	19.02
	Mid‐career	163	7.55	7.87	0.31	[0.04, 0.59]	2.23	0.026	11.66	67.48	20.86
*Overall: Pre‐placement* < *8*											
	Early career	74	4.92	6.09	1.18	[0.66, 1.69]	4.55	< 0.001	9.46	54.05	36.49
	Mid‐career	69	5.32	6.57	1.25	[0.75, 1.74]	5.02	< 0.001	7.24	49.28	43.48
*By rural/metro background*											
	Rural: Early career	127	7.50	7.59	0.09	[−3.14, 0.49]	0.43	0.670	19.69	61.42	18.90
	Rural: Mid‐career^a^	127	7.83	8.14	0.31	[0.02, 0.61]	2.08	0.040	11.81	68.50	19.69
	Metro: Early career	36	6.19	5.89	−0.31	[−1.08, 0.47]	−0.80	0.431	22.22	58.33	19.44
	Metro: Mid‐career	36	6.58	6.89	0.31	[−0.40, 1.01]	0.88	0.388	11.11	63.89	25.00
*By rural/metro background:Pre‐placement < 8*											
	Rural: Early career	50	4.92	6.52	1.60	[0.96, 2.24]	5.00	< 0.001	6.00	54.00	40.00
	Rural: Mid‐career	45	5.27	6.76	1.49	[0.91, 2.07]	5.19	< 0.001	6.67	46.67	46.67
	Metro: Early career	24	4.92	5.21	0.29	[−0.51, 1.09]	0.75	0.460	16.67	54.17	29.17
	Metro: Mid‐career	24	5.42	6.21	0.79	[−0.16, 1.75]	1.71	0.100	8.33	54.17	37.50

^a^ Wilcoxon signed‐rank result differed: W = 4109.5, *z* = 1.64, *p* = 0.101.

### 5.2 What Are the Differences for City‐ and Rural‐Background Students?

Differences among rural and city background students were examined overall and then restricted to those with scores below the overall median. Overall, there was no significant change in early‐career attraction among either rural (pre– M = 7.50, SD = 2.52, post– M = 7.59, SD = 2.30) or city background (pre– M = 6.19, SD = 2.33, post‐ M = 5.89, SD = 2.61) groups. Between‐group t–tests found rural background students had significantly higher scores pre– and post‐placement for both early‐career and mid‐career ratings, and accounted for almost all of the pre‐placement top‐scores across the early‐career (97.5%) and mid‐career (93.3%) measures.

The results showed the mid‐career mean change in attraction scores was similar but non‐significant for rural (pre– M = 7.83, SD = 2.27, post– M = 8.14, SD = 1.91) and city (pre– M = 6.58, SD = 2.06, post– M = 6.89, SD = 2.17) background students. The increase among rural students was non‐significant in the non‐parametric test, with the data for this group not meeting normality assumptions. Within the groups, the post‐placement rural attraction ratings were significantly higher for early‐career than mid‐career practice among rural students *t*(126) = 3.00, *p* = 0.003, and significantly higher for mid‐career than early career among city background students *t*(35) = 2.15, *p* = 0.038.

When restricted to only those with pre‐placement scores less than 8, the pre‐placement means of rural and city background students were similar on both measures. There was a significant positive increase in mean scores for early‐ (pre‐ M = 4.92, SD = 1.95, post– M = 6.52, SD = 2.51) and mid‐career (pre– M = 5.27, SD = 1.72, post– M = 6.76, SD = 2.14) attraction among rural background students only. Notably, very few of these rural or city students scored ‘worse’. City background students with pre‐placement scores less than 8 did not significantly increase their scores on either the early‐career (pre– M = 4.92, SD = 1.72, post– M = 5.21, SD = 2.21) or mid‐career (pre– M = 5.42, SD = 1.38, post– M = 6.21, SD = 2.04) measures. However, this may be a function of the small sample size, notably for the higher post‐placement mid‐career rating which is approaching significance, *p* = 0.100.

### 5.3 What Are the Demographic and Placement Characteristics of ‘Converters’ and ‘Sustainers’?

Converters were characterised as those who, at pre‐placement, scored less than the median on the early‐career attraction scale only, and whose scores increased by two scale points or more to 8 or higher. Sustainers were characterised as those who scored 8 or above in the pre‐placement survey early‐career attraction scale and whose score stayed above 8 in the post‐placement survey and did not decrease by two scale points (i.e., did not decrease from ‘10’ to ‘8’). Distributions of placement and demographic variables were separately assessed between converters and non‐converters who scored less than 8 in the pre‐placement survey, and sustainers and non‐sustainers who scored 8 or more in the pre‐survey. A summary is provided in Table 3.

**TABLE 3:** Characteristics and defining features of the ‘sustainers’ and ‘converters’.

	Sustainers	Converters
Definition	Early career attraction score remained > 7 and did not decrease by > 2	Early career attraction score < 8, increased by 2 or more to > 7
Reference group	Those with pre‐placement score > 7	Those with pre‐placement scores < 8
Outcome N/Reference group N	63/89	18/74
% of Reference group (% of total sample)	70.79% (38.65% total sample)	24.32% (11.04% total sample)
Significant differentiating demographic characteristics from rest of reference group	–More likely to be married or in de facto relationship –More likely to be aged 30 years and older	–More likely to be aged in their 20s –More likely to be female –More likely to retrospectively strongly agree they intended to work rurally prior to placement
Significant differentiating placement characteristics from rest of reference group	– Less likely to be involved in community activities on rural placement –More likely to agree or strongly agree they had adequate educational resources on placement	–More likely to have seen friends or family while on placement –More likely to rate their placement as much better than metropolitan placements or had no previous metropolitan placement –Slightly more likely to be rural background

Chi‐square proportion tests found sustainers were more commonly married, reported having no community activities while on their rural placement, agreed or strongly agreed they had adequate educational resources, were less likely to be aged 18–21 and more likely to be aged 30+, relative to non‐sustainers. Converters were more commonly than non‐converters to be aged 22–29, to have rated their placements as better than a city placement or have had no previous metropolitan placement at all, to have strongly agreed they intended to practice rurally prior to the placement (retrospective post‐placement assessment), and to have seen friends and/or family while on placement. They were also slightly more likely to have a rural background, approaching significance at the 5% alpha level (*p* = 0.100). There were no significant differences based on placement length.

## 6 Discussion

The results of this pre‐post study demonstrate that attraction to living and working rurally was generally very high pre‐placement in this predominantly rural background, regional university student sample. The distribution of responses is further evidence of a natural ceiling effect occurring among a substantial proportion of rural‐background students whereby attitudes to living and working rurally are unlikely to be positively changed by a placement experience. The results also indicate the overall positive effect of a single rural placement experience is limited to mid‐career attraction. Subgroup analysis indicates this may be particularly so for city background students with lower pre‐placement scores. Rural background students with below‐median pre‐placement scores significantly increased their rural attraction ratings on both measures post‐placement. This unexpected effect, combined with the analysis on ‘converters’ indicates that rural placements may serve a converting effect for more mobile rurally‐based students who are undecided about whether they will work when they graduate.

Comparisons within groups post‐placement also found that rural‐background students were more attracted to rural practice at early– rather than mid‐career, while the inverse was found for city‐background students. This, along with the large number of unchanged ratings, indicates that while many rural students are sure of staying rural when they graduate, there are many city‐background students who are also sure of staying in city areas. This is problematic as Playford and colleagues' [25] study demonstrated early‐career work location is associated with long‐term rural practice. Similarly, work by McGrail and colleagues [28] found that the period post‐medical school was an important period for career decisions associated with rural practice.

These results should be considered in an Australian inter‐regional migration context of increased immobility and greater place attachment [29, 30], with recent increases in regional mobility largely limited to coastal areas and fringes [31]. In Australia, allied health and nursing students generally move from being students to practising instantaneously with inconsistent workplace‐specific transition programs separated from their tertiary study [32, 33]. We therefore echo a recommendation from the KBC Australia Evaluation of the RHMT Program, to ‘facilitate transition of allied health and nursing students in rural, remote and regional areas’ ([5], Recommendation 28), to more directly realise RHMT Program aims.

While the converters in our sample were more likely to be from a rural background, we also note they were more likely to have met with friends or family while on placement, emphasising the importance of supporting social connections in rural areas for younger, more mobile health students to positively influence their consideration of rural practice. This adds weight to calls regarding a key overlooked long‐term rural training opportunity for nursing and allied health students [28, 34, 35], and which is known to be the main factor in the regional mobility of Australians in their 20s [36]: the decision to move to study at university. Research on the positive effects of cumulative or greater rural placement experiences has generally not considered what factors facilitate or encourage city‐background students to engage in multiple rural placements. City students studying at a regional university campus will likely have concomitantly enhanced capacity to go on rural placements and increased social ties with people from surrounding local areas. This may be a key factor in their placement experiences converting their views on the viability of working rurally in the future.

These findings are consistent with the recent large cross‐sectional UDRH collaborative study which found studying outside city universities was associated with higher pre‐placement rural practice intention among city background students, and that rural placements more positively affected city‐background students [21]. However, there is no current funding under the RHMT Program model for non‐medical professions to promote on‐campus study in regional and rural campuses to city students [4]. Such an approach would build on existing regional infrastructure, support students to gain important ties to non‐metropolitan communities, better enable travel to surrounding rural areas for placements and thereby also support cumulative rural placement experiences [10, 28]. This is a major gap in current resourcing which needs urgent attention.

From a sustaining perspective, supporting rural‐background students to remain in or near their communities for university study and placements should also be a priority [9, 37, 38, 39]. Supporting collaborative study models, such as aligning Regional University Study Hubs with proximate regional and rural university campuses and UDRHs [40], may provide students with an off‐campus study community to support engagement and progress through health study in rural areas [9, 35].

## 7 Limitations

A key limitation of this research is reflective of a key limitation of the RHMT Program: we are studying an intervention that is designed to increase the multidisciplinary rural health workforce *indirectly*. There are limitations associated with asking students about future work choices, particularly those they will make 15 years in the future due to the circumstances, preferences, and opportunities that will change during their lives. We used the term ‘attraction’ in an attempt to mitigate ceiling effects. While this may have contributed to a moderately enhanced distribution of responses, it is a more indirect measure than that of ‘intention’ that we also assume reflects future behaviour. We also had limited capacity to conduct validation checks (such as through open‐ended responses) to ensure that students have correctly interpreted the scale direction, and so we cannot rule out that some observed differences are unintentional but assume that if this occurred, it was at random. Given Australians are increasingly immobile [29], with regional migration restricted to city fringes and coastal areas [31], future research should explore rural practices in terms of where students consider their home, and distinguish increased interest in rural practice, increased intention to practice rurally, and whether likely location of practice has changed because of placement experiences.

Finally, the sample was largely from a rural background and from a regional university, with a small subsample of city‐background students. There was limited variability in the satisfaction ratings of placement experiences and supervision. Generalisability may also be limited by the high variability in health disciplines and placement length and the unique context of the rural locations included in this study. Notably, many of the placement experiences were of a relatively short duration. Comparisons of mean attraction ratings between the included sample and those who responded to only the pre‐ or post‐placement surveys found no significant differences to indicate any sample bias.

## 8 Conclusion

This study has demonstrated that the direct effect of a single rural placement experience on rural attraction is small, and for city‐background students, concentrated at the mid‐career stage and therefore potentially unlikely to occur. This is partly attributable to the sample characteristics, which showed natural ceiling effects in rural practice attraction among rural‐background students who were mostly studying at a regional university. This suggests that RHMT activities should more directly target when mobility decisions are being made (university choice and graduate work location), and consider flexible rural placement arrangements to sustain rural students' high rural practice intentions. A broader approach is needed to ensure the benefits of high‐quality rural placement experiences, and the RHMT Program, are being fully leveraged.

## Author Contributions

**Claire Ellen Seaman:** conceptualization, methodology, data curation, formal analysis, visualization, writing – review and editing, writing – original draft. **Elyce Green:** conceptualization, methodology, writing – review and editing, writing – original draft.

## Funding

This work was supported by the Australian Government.

## Disclosure

The authors note they are employed in a University Department of Rural Health at a regional university. The authors declare no other potential conflicts of interest with respect to the research, authorship, and/or publication of this article.

## Ethics Statement

This project received Charles Sturt University Human Research Ethics Committee approval, project number H18189.

## Conflicts of Interest

The authors note they are employed in a University Department of Rural Health at a regional university. The authors declare no other potential conflicts of interest with respect to the research, authorship, and/or publication of this article.

## Data Availability

The data that support the findings of this study are available on request from the corresponding author. The data are not publicly available due to privacy or ethical restrictions.
